# Argonaute Proteins: Why Are They So Important for the Legume–Rhizobia Symbiosis?

**DOI:** 10.3389/fpls.2019.01177

**Published:** 2019-10-03

**Authors:** Oswaldo Valdés-López, Damien Formey, Mariel C. Isidra-Arellano, Maria del Rocio Reyero-Saavedra, Tadeo F. Fernandez-Göbel, Maria del Socorro Sánchez-Correa

**Affiliations:** ^1^Laboratorio de Genómica Funcional de Leguminosas, Facultad de Estudios Superiores Iztacala, Universidad Nacional Autónoma de México, Tlalnepantla, Mexico; ^2^Centro de Ciencias Genómicas, Universidad Nacional Autónoma de México, Cuernavaca, Mexico; ^3^Posgrado en Ciencias Biológicas, Universidad Nacional Autónoma de México, Coyoacan, Mexico City, Mexico; ^4^Instituto de Fisiología y Recursos Genéticos Vegetales, Centro de Investigaciones Agropecuarias, Instituto Nacional de Tecnología Agropecuaria, Córdoba, Argentina

**Keywords:** argonaute proteins, legumes, symbiosis, microRNAs, small RNAs

## Abstract

Unlike most other land plants, legumes can fulfill their nitrogen needs through the establishment of symbioses with nitrogen-fixing soil bacteria (rhizobia). Through this symbiosis, fixed nitrogen is incorporated into the food chain. Because of this ecological relevance, the genetic mechanisms underlying the establishment of the legume–rhizobia symbiosis (LRS) have been extensively studied over the past decades. During this time, different types of regulators of this symbiosis have been discovered and characterized. A growing number of studies have demonstrated the participation of different types of small RNAs, including microRNAs, in the different stages of this symbiosis. The involvement of small RNAs also indicates that Argonaute (AGO) proteins participate in the regulation of the LRS. However, despite this obvious role, the relevance of AGO proteins in the LRS has been overlooked and understudied. Here, we discuss and hypothesize the likely participation of AGO proteins in the regulation of the different steps that enable the establishment of the LRS. We also briefly review and discuss whether rhizobial symbiosis induces DNA damages in the legume host. Understanding the different levels of LRS regulation could lead to the development of improved nitrogen fixation efficiency to enhance sustainable agriculture, thereby reducing dependence on inorganic fertilizers.

## Introduction

The symbiosis between legumes and rhizobia is of considerable ecological importance because through it, fixed nitrogen (e.g., ammonium) is incorporated into the food chain (Castro-Guerrero et al., 2016). In this context, it has been estimated that the legume–rhizobia symbiosis fixes 60 million metric tons of nitrogen worldwide (Smil, 1999). As symbiotic nitrogen fixation also plays essential roles in soil function, nutrient and water cycling, and food security, its exploitation and improvement in crop plants can promote lower input sustainable agriculture (Ferguson et al., 2019a).

To establish this symbiosis, a molecular dialogue between legumes and rhizobia is required (Venkateshwaran et al., 2013). This dialogue implies the interchange of diffusible signals, which includes legume-derived flavonoids and rhizobia-secreted lipochito-oligosaccharides (LCOs) with specific chemical decorations, named Nodulation Factors (NFs) (Dénarié et al., 1996). Upon NFs perception by the legume host, a series of molecular events is activated, enabling rhizobial infection and nodule formation (Venkateshwaran et al., 2013).

Legume–rhizobia symbiosis (LRS) is regulated at the transcriptional, posttranscriptional, and posttranslational level (Venkateshwaran et al., 2013). For instance, it has been demonstrated that the transcription factor (TF) Nodule Inception (NIN) controls rhizobial root infection, colonization, and nodule formation (Liu CW et al., 2019; Liu J et al., 2019). NIN also activates the expression of the *CLE ROOT SIGNALING1* (*CLE-RS1*) and *CLE-RS2* peptides in *Lotus japonicus* (Soyano et al., 2014). These two CLE peptides participate in the Autoregulation of Nodulation (AON) process, which limits the number of nodules (Ferguson et al., 2019b).

MicroRNAs (miRNAs), which are small regulatory RNA molecules, play a substantial role in the posttranscriptional regulation of LRS (Moran et al., 2017). For example, it has been demonstrated that miRNAs miR166 and miR169 regulate nodule development (Combier et al., 2006; Boualem et al., 2008) in *Medicago truncatula*. However, miRNAs not only regulate nodule development, but they also participate earlier in the rhizobial infection process (Bazin et al., 2012). The involvement of miRNAs, and likely other small RNAs (sRNAs), in the LRS strongly implicates the participation of Argonaute (AGO) proteins, which together form so-called RNA-induced silencing complexes (RISCs). We recently reported that AGO5 participates in the rhizobial infection process in both *Phaseolus vulgaris* (common bean) and *Glycine max* (soybean) (Reyero-Saavedra et al., 2017). Despite this evidence, the involvement of AGO proteins in LRS has been largely overlooked.

Here, we briefly recapitulate the genetic control of LRS by TFs and miRNAs. Likewise, based on the role of different small RNAs (sRNAs) and some AGO proteins in the regulation of both plant development and plant–pathogen interactions, we hypothesize the stages of this symbiosis where AGO proteins might play a role. Finally, we also discuss whether rhizobial symbiosis causes DNA damage in the legume host. By improving our understanding of the different levels of LRS regulation, we may be able to enhance symbiotic nitrogen fixation efficiency in crop legumes.

## Genetic Regulation of Legume–Rhizobia Symbiosis

NFs are detected by two LysM-type receptor kinases, named NFs Perception (NFP) and LysM-domain Receptor-Like Kinase3 (LYK3), in *M. truncatula* and NFs Receptor5 (NFR5) and NFR1 in *L. japonicus* (Limpens et al., 2003; Radutoiu et al., 2003; Arrighi et al., 2006). Both NFP/NFR5 and LYK3/NFR1 receptors have a similar structure, which includes an extracellular domain composed of three LysM domains, a transmembrane domain, and an intracellular kinase domain. These two receptors are essential for legume–rhizobial communication, and they may have evolved independently from two different ancestral receptors, which were likely involved in the perception of Mycorrization (Myc)-LCOs (De Mita et al., 2014). Myc-LCOs are signal molecules released by endomycorrhizal fungi and are required for most land plants to engage in symbiosis with these beneficial microbes (Maillet et al., 2011). Interestingly, Myc-LCOs and NFs are structurally very similar, which reinforces the hypothesis that NF receptors evolved from ancestral receptors involved in the perception of Myc-LCOs. The evolution of the NF’s extracellular domain arguably provided high specificity to the rhizobial symbiosis; it has been demonstrated that the evolution of the second LysM domain contributed to ligand binding, whereas the first LysM domain contributed to ligand specificity (De Mita et al., 2014).

Upon perception of NFs *via* the receptors NFP/NFR5 and LYK3/NFR1, a series of molecular events, including protein phosphorylation, are triggered (Broghammer et al., 2012). The phosphorylation of proteins is crucial to decipher the NFs signal. For example, one of the phosphorylated proteins playing a role in this symbiosis is 3-hydroxy-3-methylglutaryl coenzyme A reductase1 (HMGR1) (Kevei et al., 2007). HMGR1 participates in mevalonate biosynthesis, and it has been demonstrated that mevalonate is sufficient to trigger calcium oscillations in the nuclear region, also known as calcium spiking (Venkateshwaran et al., 2015). Calcium spiking is a crucial signature to establish rhizobial symbiosis. Membrane ion channel mutants, such as *L. japonicus castor* and *pollux* and the *M. truncatula* mutant that *does not make infections1* (*dmi1*), are unable to activate calcium spiking and therefore fail to nodulate (Imaizumi-Anraku et al., 2005).

**Figure 1:**
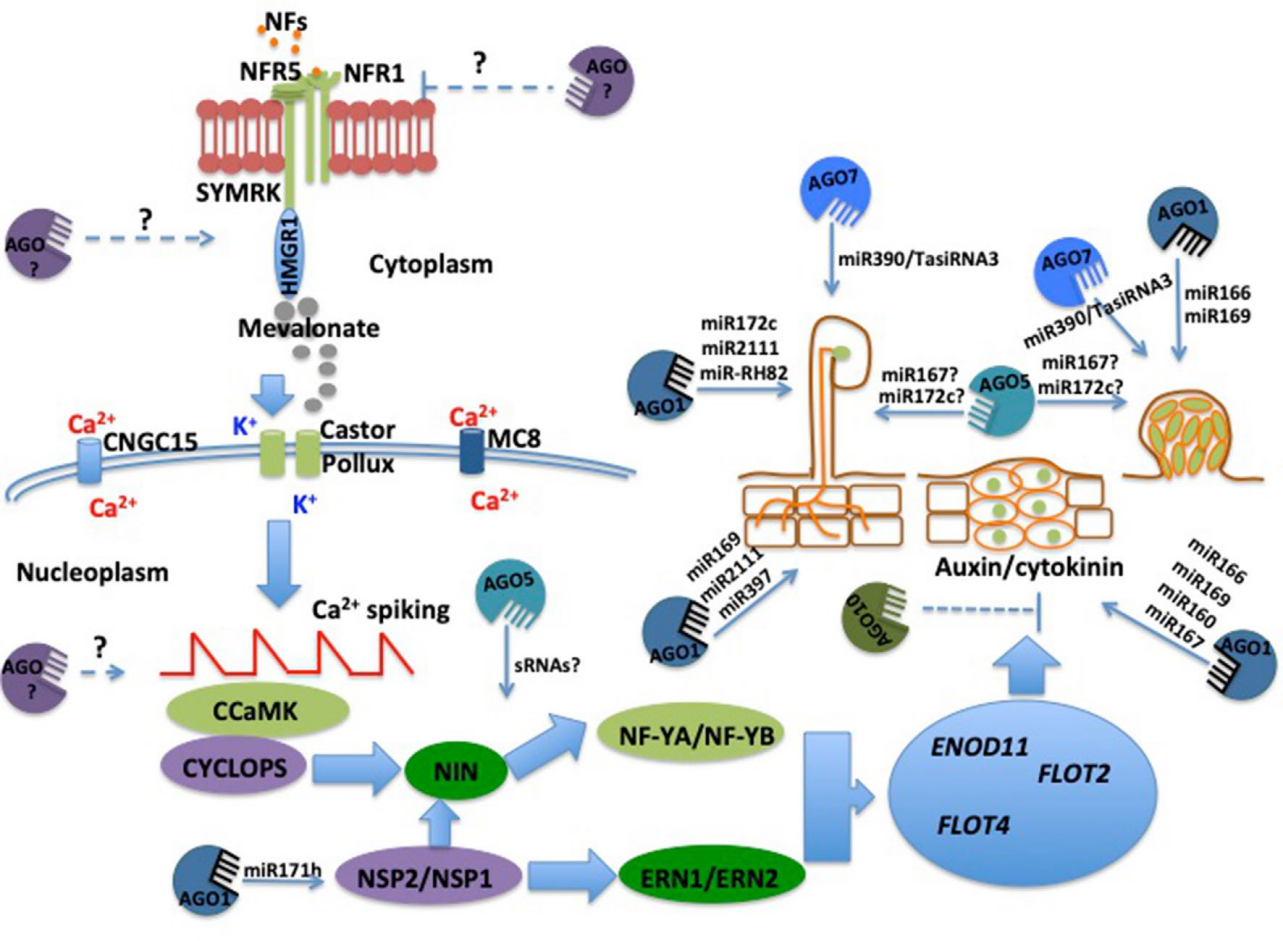
Participation of Argonaute (AGO) proteins in different stages of the legume–rhizobia symbiosis (LRS) According to several reports, different AGO proteins may participate in each stage of the LRS. Although several miRNAs have been identified a few hours upon NF perception, there is no experimental evidence indicating that they regulate very early stages of LRS, such as NFs perception and activation of calcium spiking. However, there is solid evidence supporting the participation of both sRNAs and different AGO proteins in rhizobial infection and the development of both nodule meristems and root nodules. Dashed lines indicate the potential participation of AGO proteins and sRNAs in the LRS.

Calcium spiking is further decoded by a calcium/calmodulin (Ca^+2^/CaM)-dependent protein kinase (CCaMK/DMI3) (Lévy et al., 2004). Upon activation, CCaMK/DMI3 immediately phosphorylates the transcriptional activator Interacting Protein of DMI3 (IPD3)/CYCLOPS (Singh et al., 2014). In turn, IPD3/CYCLOPS activates the expression of *NIN*, which subsequently promotes the expression of the *Nuclear Factor Y* (*NF-Y*) complexes *NF-YA* and *NF-YB* (Soyano et al., 2013). The coordinated action of these TFs and the interplay of the TF Nodulation Signaling Pathway2 (NSP2)/NSP1, Ethylene Response Factor Required for Nodulation1 (ERN1), and ERN2 lead to the transcriptional activation of symbiosis-related genes participating in the rhizobial infection process (Genre and Russo, 2016). Some of the genes activated by this transcriptional node are *Early Nodulin11* (*ENOD11*), which is involved in the infection processes (Journet et al., 2001), and the *Flotillins* (*FLOT*) *FLOT2* and *FLOT4*, which are involved in the formation of the infection thread, a tubular structure essential for rhizobial infection of the root cells (Haney and Long, 2010) (**Figure 1**).

In parallel with the activation of the molecular events leading to the infection/colonization of the root by the rhizobia, legumes activate a second genetic program that is required for nodule development (Oldroyd et al., 2011; Plet et al., 2011). It has been demonstrated that a delicate balance between the phytohormones auxin and cytokinin activates this genetic program (Hirch et al., 1989; Van Zeijl et al., 2015; Gamas et al., 2017). The activation of this genetic program begins with the inhibition of polar auxin transport, which leads to the accumulation of cytokinins in root cortical cells (Nadzieja et al., 2018). Cytokinins are then detected in root cortical cells through the receptor Cytokinin Response1 (CRE1)/Lotus Histidine Kinase1 (LHK1) (Plet et al., 2011). Interestingly, upon cytokinin perception, NIN and NSP2/NSP1 are also activated, controlling the expression of genes involved in the development of the nodule (Madsen et al., 2010).

Although this symbiosis provides fixed nitrogen to the plant, this process demands a significant amount of energy from legumes. Because of this carbon demand, legumes tightly regulate the number of nodules via AON. In *L. japonicus*, AON is systemically regulated by the CLE-RS1 and CLE-RS2 peptides (Soyano et al., 2013; Ferguson et al., 2019b). These two CLE peptides travel from the root to the shoots where they are detected by the receptor Hypernodulation Aberrant Root formation1 (HAR1) (Nishimura et al., 2002). Upon perception of CLE peptides, a signal molecule, likely a shoot-derived cytokinin or the miRNA miR2111, is produced and sent to the roots (Tsikou et al., 2018; Ferguson et al., 2019b). The perception of this shoot-derived molecule in the roots then triggers the inhibition of nodule development.

## Role of miRNAs in the Establishment of the Legume–Rhizobia Symbiosis

The first miRNAs known to be involved in the LRS were miR169 and miR166, which regulate meristem maintenance, bacterial release, and vascular differentiation in both roots and nodules of *M. truncatula* plants (Combier et al., 2006; Boualem et al., 2008). MiR169 and miR166 regulate these stages of LRS through the modulation of the expression of the TF genes *NF-YA1* (formerly called *HAP2-1* for *HAPLESS2-1*) and *class-III homeodomain-leucin zipper* (*HD-ZIPIII*), respectively (Combier et al., 2006; Boualem et al., 2008; Laloum et al., 2013). Since the publication of these two studies, a large number of symbiosis-responsive miRNAs has been identified in different stages of LRS. For instance, Subramanian et al. (2008) reported many miRNAs that were differentially regulated after 3 h of rhizobial inoculation in soybean (**Table 1**).

**Table 1:** Differentially regulated plant miRNAs and their corresponding target genes during the early stages of the legume–rhizobia symbiosis.

Reference	hpi	Regulation	miRNA	Target Gene Name
Subramanian et al., 2008	3	Up	miR168	*Argonaute 1*
			miR172	*Apetala 2 like*
			miR159	*Auxin Responsive Factor like*
			miR393	*Transport Inhibitor Response 1*
		Down	miR160	*Auxin Responsive Factor 10,16,17*
			miR164	*NAC domain containing protein 1*
			miR166	*Class III homeodomain leucine zipper*
			miR169	*Nuclear Factor YA-1 *(*Hapless 2-1*)
			miR396	*Growth-Regulating Factors*
Formey et al., 2016	6	Up	miR171a	*Nodulation-signaling pathway 2*
			miR398b-3p	*Cu/Zn Superoxide Dismutase 1/Nodulin 19*
		Down	miR171a-3p	*GRAS family transcription factor*
			miR398c	ND
			miR482b-3p	*Nucleotide-Binding Site–Leucine-Rich Repeat*
			miR-RH82	ND
*hpi, hours postinoculation; ND, nondetermined target gene.*				

Because LRS is initiated in root hairs, Formey et al. (2016) hypothesized that root hair miRNA expression analysis after 6 h of NFs treatment could identify regulators of early events of rhizobial infection. As a result, Formey et al. (2016) identified six symbiosis-responsive miRNAs in the common bean. Interestingly, one of the identified miRNAs was the root hair-specific miR-RH82. This observation suggests that this novel miRNA might play an essential role in the early stages of the LRS (Formey et al., 2016).

Although several studies report differential expression of miRNAs during the first hours of legume–rhizobia interaction, there is limited experimental evidence to indicate that they regulate very early symbiotic events, such as calcium spiking. However, miRNAs have been identified that participate in rhizobial preinfection and infection processes, including miR171c and miR397 in *L. japonicus* (**Figure 1**) (De Luis et al., 2012). Interestingly, miR171c has been shown to target transcripts of the TF gene *NSP2*, which is crucial for the preinfection and infection process (De Luis et al., 2012). To provide evidence supporting the role of these two miRNAs in the rhizobial infection process, De Luis et al. (2012) made use of *L. japonicus snf1* and *snf2* mutants, which produce autoactive versions of the CCaMK and the cytokinin receptor LHK1, respectively. These two mutants can develop nodules in the absence of rhizobia (spontaneous nodules), but they also form infected functional nodules upon rhizobial inoculation (Tirichine et al., 2006a; Tirichine et al., 2006b; Tirichine et al., 2007). By using these mutants, De Luis et al. (2012) demonstrated that miR171c and miR397 significantly accumulate in infected nodules of *snf* mutants but not in spontaneous nodules, suggesting that these miRNAs might play a role in the rhizobial infection process. Another early-acting miRNA is miR172c, which has been demonstrated to target transcripts of the TF gene *APETALA2-1* (*AP2-1*) and plays a role in rhizobia-induced root hair deformation in the common bean (Nova-Franco et al., 2015). In addition, miR172c has also been characterized in soybean, where it acts as a regulator of early nodulins during nodule initiation through the TF Nodule Number Control1 (GmNNC1) (Wang et al., 2014). In the context of the systemic AON mechanism activated upon rhizobial infection, one candidate for the induced shoot-derived inhibitor (SDI) of nodulation could be miR2111, which targets transcripts of the *F-box* gene *Too Much Love*, a crucial regulator of rhizobial infection and nodule number in *L. japonicus* (Tsikou et al., 2018; Ferguson et al., 2019b).

Moving beyond the early stages of infection, several miRNAs participating in nodule development have been reported. To initiate the formation of the nodule meristem and nodule, a delicate balance between auxin and cytokinin is required (Oldroyd et al., 2011; Plet et al., 2011). In soybean plants, miR160 is essential to modulate the levels of these two phytohormones for nodule development (Turner et al., 2013; Nizampatnam et al., 2015). Recently, it has also been demonstrated that the miR390/Trans-Actin Short Interference RNA3 module negatively regulates both rhizobial infection and nodule organogenesis in *M. truncatula* (Hobecker et al., 2017).

## Argonaute Proteins in Symbiosis

**Figure 2:**
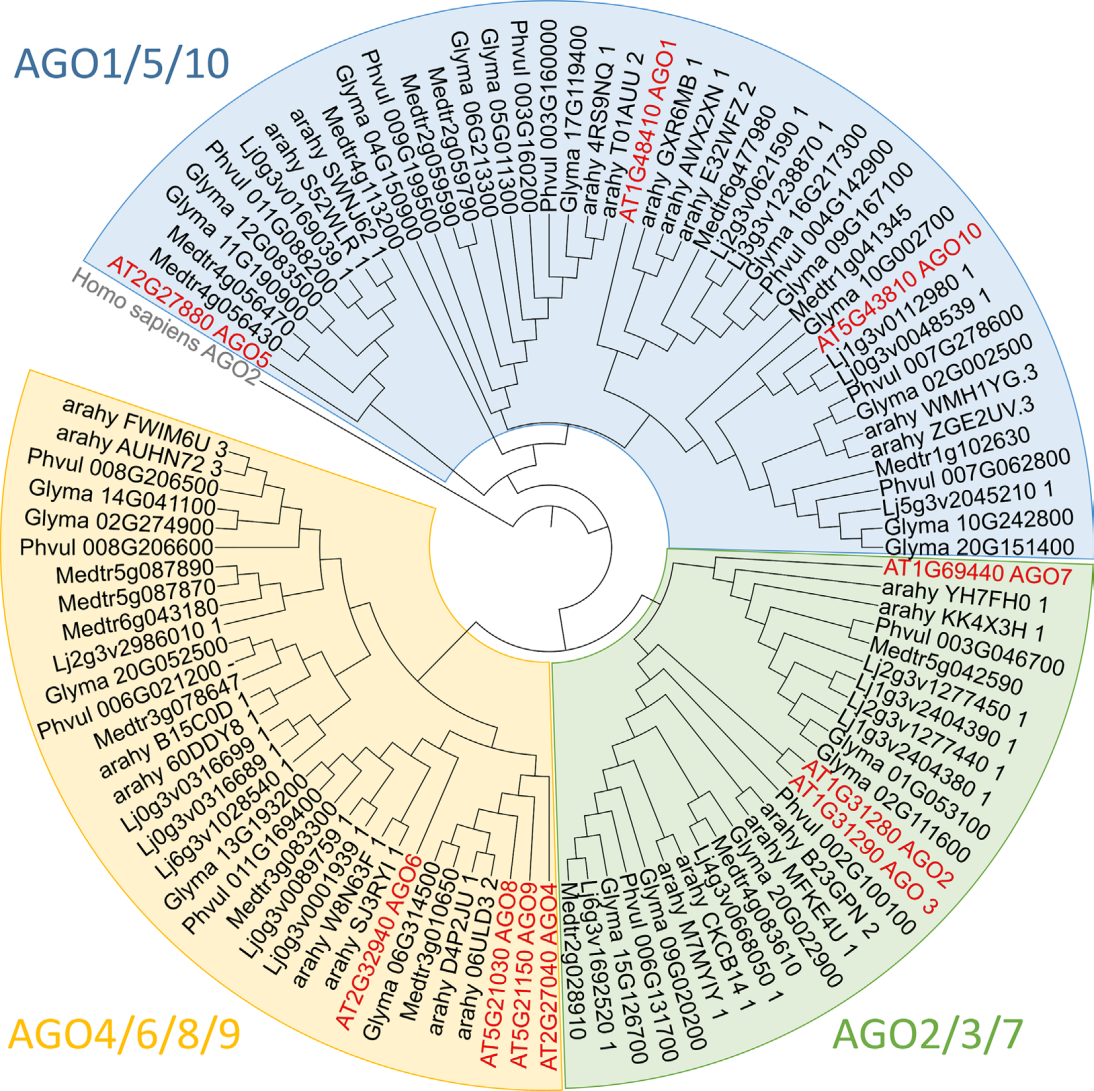
Phylogenetic analysis of legume Argonaute (AGO) family proteins The protein sequences of selected AGOs were obtained from JGI Phytozome v. 12.1.6 (https://phytozome.jgi.doe.gov), Lotus Base (https://lotus.au.dk), and PeanutBase (https://peanutbase.org) and aligned using MAFFT online service v7.427 (Katoh et al., 2017) with FFT-NS-i option set. The phylogenetic tree was constructed using the average linkage (UPGMA) method and designed thanks to iTOL 4.4.2. Abbreviations for selected species are as follows: Medtr, *Medicago truncatula*; Lj, *Lotus japonicus*; Glyma, *Glycine max*; Phvul, *Phaseolus vulgaris*; arahy, *Arachis hypgaea*; AT, *Arabidopsis thaliana*.

AGO proteins are present in eukaryotes, and they participate in many biological processes, including interactions with the environment. AGO proteins are characterized by the presence of four domains: a variable N-terminal domain and conserved PAZ (PIWI-ARGONAUTE-ZWILLE), MID (middle), and PIWI domains (Tolia and Joshua-Tor, 2007). The PAZ domain binds sRNAs, whereas the MID domain specifically recognizes the 5’ nucleotide of sRNAs. The PIWI domain adopts an RNase H-like fold, enabling most AGO proteins to cleave target messenger RNAs complementary to the bound sRNAs (Song et al., 2004). The number of AGO proteins present in plants is variable and is plant species-dependent (**Figure 2**). For instance, the *Arabidopsis thaliana* genome encodes 10 AGO proteins, whereas the soybean and the common bean genomes encode 22 and 14 AGO proteins, respectively (Liu et al., 2014; Reyero-Saavedra et al., 2017). Despite this diversity of AGO proteins in flowering plants, these proteins can be grouped into three major phylogenetic clades: AGO1/5/10, AGO2/3/7, and AGO4/6/8/9 (**Figure 2**), with AGO1 being the founding member of the *AGO* gene family (Zhang et al., 2015).

Recent evidence indicates that AGO proteins respond to environmental stimuli (Manavella et al., 2019). The direct involvement of plant AGO proteins in biotic interactions is also well known, mainly for plant defense against bacteria and virus (Raja et al., 2008; Carbonell and Carrigton, 2015; Fátyol et al., 2016). However, the role of AGO proteins in the regulation of mutualistic interactions, such as symbiosis, in animals as well as plants is poorly documented. In plants, there is only one report on this topic, which demonstrates the importance of AGO5 in LRS regulation (Reyero-Saavedra et al., 2017). Despite these knowledge gaps, several studies provide evidence that converges on the importance of AGO proteins in LRS. In this section, we aim to compile exhaustive information about legume AGO protein clades and hypothesize roles for some of them in each stage of LRS.

### Ago1/5/10 Clade

As many miRNAs have been reported as regulators of different stages of LRS (**Table 2**), AGO1 is clearly involved in this symbiotic process. For example, very recently, it was demonstrated that soybean AGO1 is hijacked by three rhizobial tRNA-derived small RNA fragments to regulate the expression of three plant genes involved in both rhizobial infection and nodule development (Ren et al., 2019). Other members of the AGO1/5/10 clade may also be involved in LRS regulation. The clearest evidence of AGO regulation of nodulation involves *AGO5* (Reyero-Saavedra et al., 2017), which is upregulated 3 h after rhizobial inoculation in common bean and soybean roots. Furthermore, AGO5 is required for rhizobia-induced root hair deformation and nodule development (Reyero-Saavedra et al., 2017). One possible explanation for this comes from *A. thaliana*, in which AGO5 associates with miR167 and miR172c (Mi et al., 2008). In legumes, miR167 and miR172c have been shown to participate in nodule development through the regulation of the *AUXIN RESPONSE FACTOR8* and *AP2-1* genes, respectively (Nova-Franco et al., 2015; Wang et al., 2015). Beyond the well-studied AGO1 protein, AGO5 is the first member of the AGO family that has been demonstrated as a regulator of LRS (Reyero-Saavedra et al., 2017).

**Table 2:** MiRNAs and Argonaute (AGO) proteins participating in different stages of the legume–rhizobia symbiosis (LRS)

miRNA	Associated AGO protein	Target Gene	Function in LRS	Legume Species	Reference
miR172c	AGO1/5	*AP2-1; NNC1*	Root hair deformation	*Phaseolus vulgaris*; *Glycine max*	Wang et al., 2014; Nova-Franco et al., 2015; Reyero-Saavedra et al., 2017
miR171c	AGO1	*NSP2*	Rhizobial infection	*Lotus japonicus*	De Luis et al., 2012
miR397	AGO1	*Laccase-Like*	Rhizobial infection	*Lotus japonicus*	De Luis et al., 2012
miR390/tasiARF	AGO7	*ARF3/4*	Rhizobial infection	*Medicago truncatula*	Allen et al., 2005
miR160	AGO1	*ARF10/16/17*	Auxins level	*Glycine max*	Turner et al., 2013; Nizampatnam et al., 2015
miR166	AGO1/10	*HD-ZIPIII*	Nodule development	*Medicago truncatula*	Boualem et al., 2008
miR167	AGO1/5	*ARF8*	Nodule development	*Phaseolus vulgaris; Glycine max*	Reyero-Saavedra et al., 2017
miR169	AGO1	*NF-YA1 (HAP2-1)*	Nodule development	*Medicago truncatula*	Combier et al., 2006

AGO10 may also be implicated in the regulation of LRS. It has been reported that AGO10 is capable of sequestering small RNAs, which consequently are not able to associate with their usual corresponding AGO family member (Zhu et al., 2011). This mechanism is involved in regulating the shoot apical meristem (SAM) in *Arabidopsis* (Zhou et al., 2015). To promote SAM differentiation, the action of miR166/165 on their target, which encodes the HD-ZIP III transcription factor, must be suppressed. To achieve this control, plants have selected a regulation system based on the sequestration of miR165/166 by AGO10, which has a higher affinity for these miRNAs than AGO1 and can promote their degradation (Yu et al., 2017). Although this mechanism has not been demonstrated directly in root apical meristem differentiation, some evidence suggests that it could be involved (Ma et al., 2017). In addition, the AGO10 regulatory mechanism is considered an ancient and ubiquitous process in land plant organ development. In *M. truncatula*, the miR166/*HD-ZIP III* node regulates both lateral root and nodule formation through the control of the apical region (Boualem et al., 2008). If the regulation of miR166/*HD-ZIP III* node by AGO10 proteins is a general mechanism, it is tempting to speculate that nodule development could also be controlled in this way. In support of this possible role in LRS, transcripts of *AGO10* group member genes in *M. truncatula*, *Glycine max*, and *P. vulgaris* are upregulated in nodules compared to root tissues (Phytozome v. 12.1.6). This reinforces the hypothesis that AGO10 could be a player in the regulation of nodule development.

### AGO2/3/7 Clade

Beyond the phylogenetic grouping, members of the *AGO2/3/7* clade seem to be connected by an involvement in plant defense, employing different regulation mechanisms (Zhang et al., 2011;Fang and Qi, 2016; Rodríguez-Leal et al., 2016). Because AGO2 and AGO3 members are difficult to distinguish in legumes, due to the phylogenetically clustering of the two members by species of origin and not by member type (Zhang et al., 2015), here we focus on the “AGO2/3” group and AGO7 (**Figure 2**).

In *A. thaliana*, AGO2 is a key player in both antiviral defense and antibacterial immune response (Zhang et al., 2011; Carbonell and Carrigton, 2015). Moreover, AGO2 is the only member of the *A. thaliana* AGO family reported as highly induced during *Pseudomonas syringae* infection (Zhang et al., 2011). AGO2 acts in this process by loading miR393b*, which targets transcripts of the gene *MEMB12* encoding a Golgi-localized, SDS-resistant, soluble N-ethylmaleimide-sensitive factor attachment protein receptor (SNARE), and then modulates the exocytosis of antimicrobial Pathogenesis-Related (PR) proteins. LRS is intimately linked to plant immunity (Toth and Stacey, 2015), and PR proteins seem to regulate the rhizobial infection process in soybean and *L. japonicus* (Bartsev et al., 2004; Hayashi et al., 2014). In this context, the involvement of AGO2/3 in the regulation of LRS should be considered. Supporting this hypothesis, AGO2/3 homologs in *M. truncatula*, *G. max*, and *P. vulgaris* are upregulated in nodules compared to root tissues (Phytozome v. 12.1.6). In addition, analysis of legumes AGO proteins shows that AGO2 has undergone gene duplication in *M. truncatula*, *G. max*, and *L. japonicus* (Bustos-Sanmamed et al., 2013) (**Figure 2**). This gene duplication of AGO2/3 suggests that the AGO2/3 isoforms may have diverged in their biological function and could be involved in novel processes, including LRS regulation.

AGO3 is one of the least studied members of the AGO family in plants and, to date, poor information is available about its functionality. Minoia and collaborators (2014) revealed that AGO3 binds siRNAs derived from potato spindle tuber viroid and could be involved in the defense against this pathogen. Similarly, in a recent preprint, Jullien and collaborators (2018) suggest a role of AGO3 in antiviral defense based on its confinement to vascular structures and the fact that most plant viruses use the phloem for systemic infection. However, further analyses are needed to understand the role of this AGO member and confirm its role in plant antiviral response. At this time, the link between AGO3 and the plant–microorganism interaction is speculative.

AGO7 is involved in the biogenesis and actions of trans-acting small interference RNAs (tasiRNAs, also called phasiRNAs), which are plant-specific endogenous siRNAs derived from long double-stranded RNA, and participate in plant development (Adenot et al., 2006). AGO7 also plays a critical role in the regulation of both plant immunity and antiviral defense (Adenot et al., 2006; Carbonell and Carrigton, 2015). For example, AGO7 is also essential for the generation of the bacteria-induced small RNAs called long small interfering RNAs (lsiRNAs) (Katiyar-Agarwal et al., 2007). AtlsiRNA-1 is induced by bacterial pathogens and participates in plant resistance by silencing *AtRAP*, which encodes a RAP-domain protein involved in plant defense (Katiyar-Agarwal et al., 2007). This regulatory role of AGO7 in pathogen response mechanisms could be modulated to contribute to the fine-tuning of plant bacterial resistance under LRS. In support of this, *P. vulgaris* AGO7 is upregulated upon inoculation with rhizobia deficient in the production of NFs or lipopolysaccharides (Dalla Via et al., 2015), which are symbiotic signals able to suppress the plant defense response during symbiosis (Albus et al., 2001; Scheidle et al., 2004). Besides, mutation of the *AtAGO7* homolog in *L. japonicus* and *M. truncatula* reduces rhizobial infection and nodule number compared to the corresponding wild type (Li et al., 2014; Hobecker et al., 2017). Part of this response is also possibly due to the capacity of AGO7 to generate secondary small RNAs derived from the miR390-induced degradation of the *TAS3* transcript (Allen et al., 2005). The derived tasiRNAs target the *ARF2*, *3* and *4* gene transcripts. These ARF TFs control part of the auxin signaling pathway, which also plays a key role in LRS (Breakspear et al., 2014).

### AGO4/6/8/9 Clade

The AGO4/6/8/9 protein clade is oriented toward transcriptional regulation by DNA methylation (Mallory and Vaucheret, 2010; Duan et al., 2015). In legumes, this clade differs from other families. In *G. max*, *L. japonicus*, *M. truncatula*, and *P. vulgaris*, AGO4 and 6 are present but not AGO8. In the case of AGO9, this protein is absent in most legumes, except in *G. max*. This loss of diversity for the AGO8/9 group in legumes is compensated by the diversification of AGO4, which displays between two and four isoforms in the genome of model legumes (Bustos-Sanmamed et al., 2013) (**Figure 2**). This specific legume pool of AGO4 isoforms is phylogenetically separated from nonlegume AGO4, suggesting specialization in legumes. Supporting this hypothesis, in *G. max, M. trucatula*, and *P. vulgaris*, at least one of the AGO4 isoforms is differentially accumulated in nodules compared to root tissues, which suggests that this AGO4 isoform might play a role in the LRS (Phytozome v. 12.1.6).

## Does Rhizobial Symbiosis Cause Damage in the Legume DNA?

Several studies have reported that plant pathogens can trigger damage in the host plant DNA (e.g., DNA double-strand breaks) (Song and Bent, 2014; Hadwiger and Tanaka, 2017). Some pathogen-induced DNA damage is triggered by reactive oxygen species (ROS) (Song and Bent, 2014; Hadwiger and Tanaka, 2017). It has been demonstrated that AGO2 and AGO9 play roles in DNA repair in *A. thaliana* (Wei et al., 2012;Oliver et al., 2014). Very recently, it has been reported that *Rhizobium huautlense* produces ROS in *Caenorhabditis elegans* intestinal cells, which then leads to DNA damage (Kniazeva and Ruvkun, 2019). Interestingly, during the rhizobial infection process, the production of ROS is essential for the formation of the infection thread (Damiani et al., 2016). Despite the evidence from animal cells and the fact that symbiotic rhizobia trigger ROS production, there is no experimental evidence to suggest that rhizobial symbiosis causes DNA damage in legume hosts. However, to allow rhizobial infection of the host, nodule cells undergo genome endoreduplication, often considered a protective mechanism against DNA damage to maintain whole-genome integrity (Maroti and Kondorosi, 2014). Further investigation is needed to explore whether rhizobia can cause DNA damage in legume hosts and whether AGO proteins (i.e., AGO2 and AGO9) participate in DNA repair in the context of LRS.

## Perspectives and Conclusions

Based on the participation of many different types of sRNAs, it is clear that different members of the AGO protein family might play crucial roles in LRS (**Figure 1**). However, it is still unclear how the participation of each AGO protein occurs and how it is regulated. Hence, the new challenge will be to understand how, when, and where AGO proteins are regulated during LRS. Having this knowledge will help us develop a clear idea about the relevance of AGO proteins in rhizobial symbiosis.

## Author Contributions

OV-L and DF designed the concept and organization of the manuscript. OV-L and DF wrote the manuscript with the help of MI-A, MR-S, TF-G, and MS-C.

## Funding

This work was supported by the Programa de Apoyo a Proyectos de Investigación e Inovación Tecnológica (PAPIIT grant No. IN213017) and by the Consejo Nacional de Ciencia y Tecnología (CONACyT grant No. A1-S-9454) to OV-L. This work was also partially supported by a CONACyT grant (A1-S-16129) and PAPIIT grant (IA203218) to DF. MI-A is a doctoral student from Programa de Doctorado en Ciencias Biológicas, Universidad Nacional Autónoma de México, and receives a fellowship from CONACyT (CVU: 919676). MR-S is a doctoral student from Programa de Doctorado en Ciencias Biomédicas, Universidad Nacional Autónoma de México, and receives a fellowship from CONACyT (347027/239879).

## Conflict of Interest

The authors declare that the research was conducted in the absence of any commercial or financial relationships that could be construed as a potential conflict of interest.
